# Complete mitochondrial genome of *Cnidocampa flavescens* (Lepidoptera: Limacodidae)

**DOI:** 10.1080/23802359.2017.1365651

**Published:** 2017-08-18

**Authors:** Shuying Peng, Yuan Zhang, Xiaochun Zhang, Yan Li, Zhuoran Huang, Yunfang Zhang, Xu Zhang, Jianhua Ding, Xuexia Geng, Jun Li

**Affiliations:** College of Life Science, Huaibei Normal University, Huaibei, P.R. China

**Keywords:** Mitochondrial genome, *Cnidocampa flavescens*, Lepidoptera, Limacodidae, mitogenome

## Abstract

*Cnidocampa flavescens,* lives in Nepal, Bhutan, China, Far East of Russia, Korea, and Japan, belongs to the Lepidoptera family Limacodidae. In this study, we describe the genomic features of the mitogenome sequences of the insects. The mitogenome of *C. flavescens* is 15,406 bp long consisting a typical set of genes (13 protein-coding genes, 22 tRNA genes, and 2 rRNA genes) and one major 415 bp non-coding A + T-rich region. All PCGs of *C. flavescens* start with ATN codons and end with TAA codons. The gene arrangement of *C. flavescens* mitogenome is same to *Monema flavescens* while the intergenic spacers and overlaps are different. The 415 bp A + T-rich region contains a conserved ATAGA motif followed a poly-T stretch.

*Cnidocampa flavescens* (Walker) belongs to Lepidoptera family Limacodidae. They have been found in in Nepal, Bhutan, China, Far East of Russia, Korea, and Japan (Pan et al. 2013). Mitogenomes have been involved in diverse studies of molecular evolution for its protein-coding genes (PCGs), sequence conservation, maternal inheritance, and rapid evolution. And only one complete mitochondrial genome of Limacodidae has been reported (Liu et al. 2016).

The specimens were obtained from Xiangshan Mountain, Huaibei City, Anhui Province, P.R. China (116°48′34″E, 33°59′1″N). We extracted the total DNA from the specimens as PCR template, designed PCR primers according to the sequence conservation of mitochondrial DNA of Lepidoptera insects, amplified the overlapping fragments using high fidelity Taq enzyme, and ensured every fragments sequenced at least three times. These fragments were assembled in a complete linear mitochondria DNA sequence using the DNAStar package (DNASTAR Inc. Madison, WI) and the mitogenome annotated using NCBI BLAST (http://blast.ncbi.nlm.nih.gov/Blast).

Multiple sequence alignments were conducted using Clustal X 2.1, and phylogenetic trees were constructed based on complete mitochondrial DNA sequences by MEGA 6.06, using the maximum-likelihood method with a bootstrap test of 1000 replications. Two species of Lasiocampoidea were utilized as out-group.

The complete mitogenome of *C. flavescens* (GenBank accession number KY628213) is 15,406 bp and consists of 13 PCGs, 22 tRNAs, 2 rRNAs for the small and large subunits (rrnS and rrnL), and 1 AT-rich region (control region). All PCGs start with ATN codons and stop at TAA codons, which are somewhat different from *Monema flavescens*.

The gene arrangement of *C. flavescens* mitogenome is same to *M. flavescens.* But the intergenic spacers and overlaps are different. Eighteen intergenic spacers (345 bp in total) were founded in *C. flavescens* mitogenome. The intergenic nucleotides vary from 1 to 97 bp and the longest located between *trnQ* and *nad2*. Though the longest intergenic spacer of *M. flavescens* also located between *trnQ* and *nad2,* the intergenic nucleotides are 50 bp and less than that of *C. flavescens* (Liu et al. 2016). The overlap nucleotides of *C. flavescens* vary from 1 to 20 bp and only one overlap longer than 9 bp is between *cox2* and *trnk*, while the overlap nucleotides of *M. flavescens* are not more than 9 bp (Liu et al. 2016) (Figure 1).

**Figure 1.**
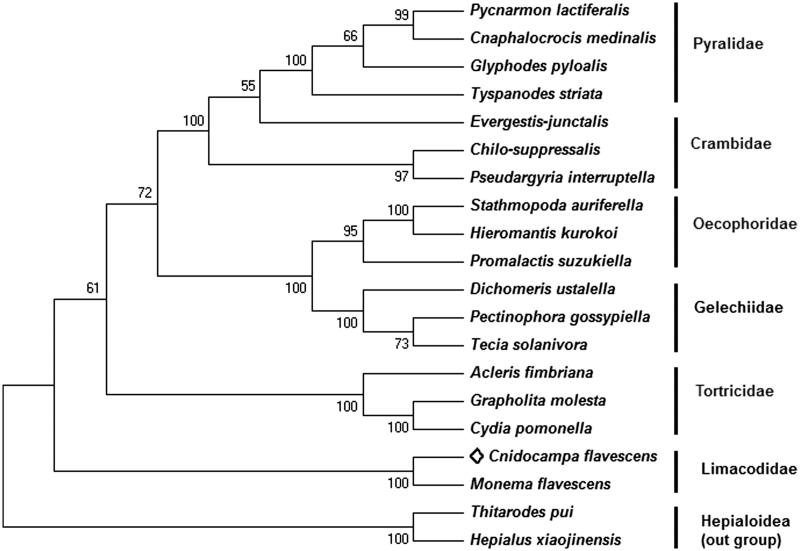
Phylogenetic tree constructed by the maximum-likelihood (ML) method with a bootstrap test of 1000 replications based on complete mitochondrial DNA sequences. Two species of Hepialoidea were utilized as out-group. GenBank accession numbers are as follows: *C. flavescens* KY628213 (the present study); *Pycnarmon lactiferalis* KX426346 (Chen et al. 2016); *Cnaphalocrocis medinalis* JQ647917 (Wan et al. 2013); *Glyphodes pyloalis* KM576860 (Kong and Yang 2016); *Tyspanodes striata* KP347977 (unpublished); *Evergestis junctalis* KP347976 (unpublished); *Chilo suppressalis* JF339041 (Chai et al. 2012); *Pseudargyria interruptella* KP071469 (Song et al. 2016); *Stathmopoda auriferella* KX138529 (Jeong et al. 2016); *Hieromantis kurokoi* KU605775 (Park, Jeong, Kim, et al. 2016); *Promalactis suzukiella* KM875542 (Park, Kim, Kim, et al. 2016); *Dichomeris ustalella* KU366706 (Park, Kim, Jeong, et al. 2016); *Pectinophora gossypiella* KM225795 (Zhao, Sun, et al. 2016); *Tecia solanivora* KT326187 (Ramirez-Rios et al. 2016); *Acleris fimbriana* HQ662522 (Zhao, Wu, et al. 2016); *Grapholita molesta* HQ392511 (Gong et al. 2012); *Cydia pomonella* JX407107 (Shi et al. 2013); *Monema flavescens* KU946971 (Liu et al. 2016); *Thitarodes pui* KF908880 (Yi et al. 2016); *Hepialus xiaojinensis* KT834973 (Chen et al. 2017).

The 415 bp A + T-rich region of *C. flavescens* located between *rrns* and *trnM* contains an ATAGA motif, followed by a poly-T stretch which has Lepidoptera-specific features. At the end of the A + T-rich region, there is also a poly-A stretch just like *M. flavescens* (data not shown).

## Depository

The specimens are deposited in the specimen room and the DNA is deposited at Human and Animal Genetics Laboratory, College of Life Science, Huaibei Normal University, Huaibei City, Anhui Province, P.R. China.
